# 1727. Household Vaccination Status and Attitudes towards Pediatric COVID-19 Vaccination: A survey at four New Jersey clinic-based pediatric practices

**DOI:** 10.1093/ofid/ofad500.1559

**Published:** 2023-11-27

**Authors:** Bozena J Katic, Jessica L Alvitres, Uzma Hasan, Aspasia Katragkou, Stephen M Friedman, Joseph V Schwab, Alan Weller, Pauline Thomas, Mary C Kennedy, Charles Li, Manisha Gurumurthy, Claudia Rohan, Dorothy Chu, Mawada Hussein, Mehek Shaikh, Bijal Damania, Harini Morisetty, Grayland W Godfrey, Sunanda Gaur, Hathija Noor

**Affiliations:** CDC foundation, Trenton, New Jersey; New Jersey Department of Health, Trenton, New Jersey; Saint Barnabas Medical Center, West orange, New Jersey; Goryeb Children's Hospital, Atlantic Health System, Morristown, New Jersey; Rutgers New Jersey School of Medicine, Livingston, New Jersey; Rutgers New Jersey Medical School, Newark, New Jersey; Rutgers Robert Wood Johnson Medical School, Metuchen, New Jersey; Rutgers New Jersey Medical School, Newark, New Jersey; Goryeb Children's Hospital/Atlantic Health System, Morristown, New Jersey; Rutgers Robert Wood Johnson Medical School, Metuchen, New Jersey; Rutgers NJMS, Skillman, New Jersey; Atlantic Health Sysytem, Morristown, New Jersey; Cooperman Barnabas Medical Center, Livingston, New Jersey; RWJBH, Staten Island, New York; Newark Beth Israel Medical Center, Newark, New Jersey; Rutgers Health Newark Beth Israel, Newark, New Jersey; Rutgers New Jersey Medical School, Newark, New Jersey; Newark Beth Israel/ Rutgers Health, Harrison, New Jersey; Rutgers Robert Wood Johnson Medical School, Metuchen, New Jersey; Newark Beth Israel Medical Center, Newark, New Jersey

## Abstract

**Background:**

Although COVID-19 vaccination has been recommended for all children 6 months of age and older since August 2022, vaccine uptake among the pediatric population is low. Underlying caregiver attitudes towards pediatric COVID-19 vaccination have not been adequately classified or described. A better understanding of how caregiver attitudes influence household vaccination behavior can help tailor future vaccine interventions.

**Methods:**

Unvaccinated children aged 18 months to 11 years underwent antibody testing and their caregivers were asked to complete an electronic survey at four clinic-based practices in Northern and Central NJ from September 2022 to April 2023. Information was collected on household exposures, vaccination status, COVID-19 infection, and caregiver beliefs about pediatric vaccination. Latent class analyses were conducted to group caregivers into four distinct profiles based on how safe, effective, useful and necessary they felt vaccination was.

**Results:**

A total of 656 survey participants were grouped into a vaccine acceptance class based on their level of agreement on each attitudinal item, from low to high. Those in Class 1 had the most disagreement and lowest vaccine acceptance; those in Class 4 had the most agreement and highest vaccine acceptance. The majority of the sample fit into Class 2 (39%), indicating moderate disagreement and low COVID-19 vaccine acceptance.

Caregivers in the lowest vaccine acceptance classes (1 & 2) were significantly less likely to plan to vaccinate their children compared to those in higher classes (3 & 4); (9.8% and 5.5% vs. 31.9% and 61%).

Those in Class 2 were significantly less likely to have at least one adult household member fully vaccinated or boosted against COVID-19 than those in higher classes; and were significantly more likely to be from households where at least one adult was diagnosed with COVID in the past year than those in Class 3 (54.2% vs 37.7%).

**Figure:**
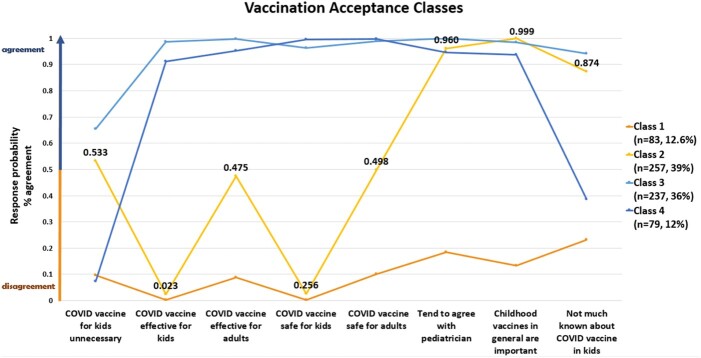

**Figure:**
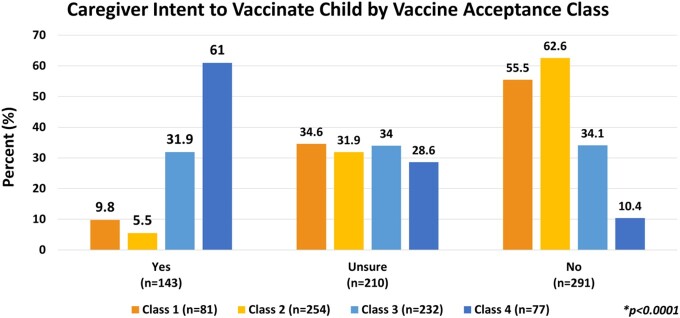

**Figure:**
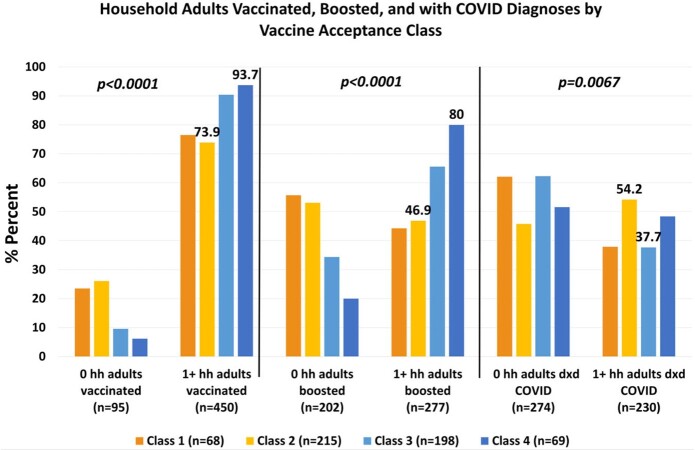

**Conclusion:**

Caregiver attitudes towards vaccination, and inclusion in vaccine acceptance classes, is closely associated with household vaccination status and COVID-19 infection history. Educational efforts and messaging should target the different aspects of caregiver profiles to address vaccine hesitancy and make future vaccine programming for children more successful.

**Disclosures:**

**Joseph V. Schwab, MD, MPH**, Bristol-Myers Squibb: Stocks/Bonds|GE Healthcare: Stocks/Bonds|Johnson and Johnson: Stocks/Bonds

